# The Innovative Medicines Initiative −10 Years of Public-Private Collaboration

**DOI:** 10.3389/fmed.2019.00275

**Published:** 2019-12-03

**Authors:** Hugh Laverty, Pierre Meulien

**Affiliations:** ^1^Head of Scientific Operations, Innovative Medicines Initiative, Brussels, Belgium; ^2^Executive Director, Innovative Medicines Initiative, Brussels, Belgium

**Keywords:** public-private partnership, healthcare, medicines, innovation, multi-stakeholder, society, European Union, industry

## Abstract

The Innovative Medicines Initiative (IMI) is a public-private partnership between the European Union and the European pharmaceutical industry. Born of the necessity to foster collaboration between different stakeholders in order to address growing challenges in bringing new medicines to market and the rapidly evolving healthcare landscape, IMI has successfully delivered the radical collaboration needed to address these challenges. In this article we reflect on some of the major achievements of the programme by highlighting a few of the key projects funded and the progress they have made, as well as some of the lessons learnt in delivering such an ambitious partnership. Those that drove the foundation of IMI recognized that to address these challenges required not just ambitious scientific approaches, but also an awareness of societal needs. Therefore, actors from beyond the traditional pharmaceutical research communities would be needed. One of the key successes of IMI has been to foster radical collaboration between diverse public and private partners of all types, including large pharmaceutical companies, SMEs, regulators, patient organizations and public research institutions. It has achieved this by being a neutral platform where all partners are bound by the same rights and responsibilities. Since it began there has been an evolution in the understanding of what is considered “pre-competitive,” resulting in IMI projects now addressing all of the steps within the pharmaceutical development value chain. With this expansion in the types of projects supported by IMI, different actors from beyond the traditional pharmaceutical research family have been attracted to participate, enriching further the collaboration at the heart of the programme. Finally, such a complex programme brings with it challenges, and we reflect on some of the important learnings that should be applied to future collaborative models to ensure that they are as successful as possible and deliver the expected impact.

## Introduction—The Need for a Public-private Partnership in Health

It is clear that the economic sustainability of our health systems in Europe (and elsewhere) is under threat. Whether we are talking about affordable medicines, the lack of sufficient healthcare professional resources, or society's challenge in investing in prevention, all angles of the healthcare ecosystem are currently stretched (1).

Given the scale and complexity of the challenges faced, the only route by which they can be addressed is through multidisciplinary, multi-stakeholder approaches where the risks and benefits of overcoming them are shared. For the past 11 years, the Innovative Medicines Initiative (IMI) has been promoting the radical collaboration necessary to overcome some of these challenges in relation to speeding up the development of, and patient access to, innovative medicines, particularly in areas where there is an unmet medical or social need. There are 4 broad areas in which IMI projects operate; the first is where there are currently market failures i.e., no incentives for private sector investments; the second is in tackling highly complex diseases where pre-competitive consortia are necessary to accelerate knowledge acquisition to a point where product development is envisaged; the third area is providing technology platforms where private and public entities pool resources to improve drug development; and finally, addressing gaps in the drug discovery/development ecosystem where precompetitive collaboration is necessary to overcome the challenge. Information related to the setting up of IMI and progress of the programme has been published previously (2–4). In this article we reflect on how IMI is able to promote collaboration, and what could be delivered by using this collaborative model as the basis for future partnerships. We reflect on some of the challenges faced, and discuss how this collaborative model could be extended to encompass current societal challenges and deliver the changes necessary to help healthcare become more affordable and sustainable for all.

## Evolution of the IMI Programme

The Innovative Medicines Initiative Joint Undertaking (JU) is a public-private partnership (PPP) between the European Union (EU), represented by the European Commission (EC), and the European Federation of Pharmaceutical Industries and Associations (EFPIA)^1^. When it was launched in 2008, the overall goal of the first IMI programme (IMI1) was to “significantly improve the efficiency and effectiveness of the drug development process with the long-term aim that the pharmaceutical sector produces more effective and safer innovative medicines.”

In order to make progress toward this goal, a budget of EUR 2 billion was mobilized. Half of this budget came from the EU's seventh framework programme for research and innovation (FP7), which ran from 2007 to 2013. The remaining budget came from EFPIA through its member companies and associations, with the majority of support coming as of “in-kind” contributions. In kind contributions are usually in the form of the time of company researchers working on the projects and the reagents and equipment used in the projects. It is important to remember that none of the EFPIA companies receive any EU funding via IMI; the EU funding supports the participation in IMI projects of universities, research centres, small and medium-sized enterprises (SMEs), patient groups, and regulators.

The IMI1 programme focused on addressing challenges in the early pre-competitive space of pharmaceutical research and development. However, it was soon recognized that other areas that had traditionally been viewed by some as “competitive” also required collaborative approaches. Therefore, while the second phase of IMI (IMI2) is still a collaboration between the European pharmaceutical industry and the EU, the partnership has a broader scope in terms of the questions being addressed. It is also more disease specific, more open in terms of project-specific partnerships, and is able to tackle some market or scientific failures important for public health, e.g., antimicrobial resistance and Alzheimer's disease.

IMI2 operates under the EU's current framework programme for research and innovation, Horizon 2020, which runs from 2014 to 2020. IMI2 has a total budget of up to EUR 3.276 billion, half of which comes from Horizon 2020, and half of which comes from EFPIA member companies and IMI Associated Partners. In addition to mobilizing pharmaceutical companies, the legislation creating IMI2 also emphasizes the need to bring partners from other sectors (e.g., diagnostics, animal health, IT, imaging, etc.) into the IMI community. This open nature of the programme is reflected in the creation of “Associated Partner” status that allows organizations that are not EFPIA members to contribute in kind to IMI projects, and have that contribution matched by the EU. This mechanism has acted as a magnet for those partners who see the advantage of the neutral, multi-stakeholder platform that has been created through IMI.

### Building Trust With Stakeholders

Now that IMI has passed its 10^th^ year of existence some of the initial skepticism to the programme has been forgotten. When IMI was first discussed two key concerns for public institutions were the ability to publish research papers and the management of intellectual property rights (IPR). The fear was that the involvement of pharmaceutical companies would block the publication of new research findings or they would take any IPR for themselves. In both of these situations these fears have proven to be unfounded. Regarding the ability to publish, IMI undertakes a bibliometric analysis of project outputs each year and publishes the report on its website. The recent report related the period 2010-2018 has identified 4,938 publications in the Clarivate Web of Science database. The majority of these publications (60%) have been published in high impact journals i.e., those journals in the highest quartile ranked by Journal Impact Factor. The impact of IMI project research (as indexed by citation impact) remains high, with the field normalized citation impact of IMI project research of nearly 1.84 nearly twice the world average of 1.00. Given the highly collaborative nature of IMI projects, their multi-disciplinary partners and the diverse datasets involved, the high level of publication and its quality is not surprising. IMI projects are highly collaborative; 62.2% of all IMI project papers were co-authored by researchers working in different sectors, 84.3% involved collaboration between institutions, and 61.3% of all IMI project papers were internationally collaborative. Internationally collaborative IMI research has a citation impact of 2.64, well over twice the world average. Some projects such as BTCURE^2^ (IMI1 Call2) have been very prolific in publishing with 645 publications as of the end of 2018.

Another area of concern was the management of Intellectual Property (IP) and ownership of results, but again experience has shown that for many companies, tools and methods that improve the efficiency of their processes are more important than the generation of patents. The IMI IP provisions/rules^3^ govern the IP regime of all projects supported by IMI and apply equally to all partners (public and private) in the projects. The IP provisions are designed to support innovation while respecting the interests of all project partners along the following principles: the IP rights of the pre-existing assets brought to a project are preserved and are identified before the project begins as “Background,” and ownership of results generated during the project follows inventorship. The policy actually empowers the results owners to decide on the best protection modalities. However, to ensure that the project can be implemented by all the partners, basic access rights to results generated within the project are granted on an equal basis and this provides opportunities of further development and/or validation of projects results. When a project asset is mature enough, access rights for exploitation purposes can be negotiated on a case-by-case basis. IMI favors open access dissemination of project results, but this is subject to legitimate interests and therefore results that may generate value can also be protected by project partners.

An important aspect of IMI's IP provisions is their flexibility, which allows them to be adapted to the needs of an individual project and the participants. As a result, different projects can adapt the provisions to suit whether they are developing an open platform for the research community where access to the data is important, or developing late stage assets in challenging areas such as AMR where the value of those assets may need to be protected.

The importance of engaging meaningfully with patients was recognized as a key goal of IMI from its inception. The key actors in drug development such as the pharmaceutical industries and regulatory agencies have historically been perceived as being too far removed from patients and sometimes taking decisions in which patient interests are not fully reflected. Since its creation IMI has been expanding the ways in which it engages with patients. Many IMI projects engage and involve patients to ensure their experiences can be taken into account and as of the end 2018, close to 60% of ongoing IMI projects have patient organizations either as partners in the consortium or represented on advisory boards, ethics advisory boards, or being consulted for topics of relevance. There was also a conscious effort to move beyond paying lip service to patients and recognize them as full partners in the process. Several IMI projects are focused fully on ensuring that patients and their experiences are fully integrated into the drug development process, while ensuring that trust between different stakeholders is enhanced.

EUPATI^4^ focused on providing education and training support to increase the capacity and capability of patients to understand and contribute to medicines research and development. It also worked to improve the availability of objective, reliable and patient-friendly information to the public in several different European languages. The integration of patients into medicines development processes needs to be done in a way that is structured, governed by clear rules and modes of operation to be effective and yield the best results for all stakeholders. The EUPATI project has worked closely with all stakeholders to prepare overarching guidances on meaningful and ethical patient engagement for regulatory processes (5); health technology assessment (6); ethical review of clinical trials (7), and pharmaceutical industry–led medicine [R&D; (8)].

The project PARADIGM^5^ builds on these initiatives and is attempting to strengthen the understanding of stakeholders' needs and expectations for engagement (including underrepresented and vulnerable populations). In addition, they aim to build further guidance in three key decision–making points in the medicines development process, research and priority setting, clinical trial design, and early dialogues with regulatory and health technology assessment bodies. Another project focused on engaging and involving patients, PREFER^6^, will establish recommendations to support the development of guidelines for industry, regulatory authorities and HTA bodies on how and when to include patient perspectives on benefits and risks of medicinal products (9).

The IMI programme office also recognizes its responsibility toward patients and their carers and has explored how to engage better and involve them more in the work of the IMI programme. From having patient representatives on the Scientific Committee (SC, an advisory body to the IMI Governing Board, GB) to patient dedicated workshops, the IMI office has been exploring the best way to involve patients. In 2019 IMI created a patient expert pool who are called upon to provide input on IMI's scientific strategy by taking part in consultations, participating in workshops, panels to review project proposal, reviews of ongoing and closed projects, review content of materials targeted at patients and the wider public as well as participate in IMI events. All patients carry out their activities in a personal capacity and do not represent an organization. In total 157 applicants (118 patients and 39 family members/carers) have been added to the pool, with 57% female and coming from 26 countries. A large majority have knowledge of research and innovation activities. They have direct experience as patients or carers of cancer, infectious disease, inflammatory/immune diseases, metabolic disease, neurodegenerative disease, neuropsychiatric disorders and pain as well as rare and orphan diseases.

Scientific knowledge is one of the keystones of regulatory decision making and many IMI projects generate data that is of direct relevance to regulatory authorities, health technology assessment (HTA) bodies and payers. Experience to date has shown that regulatory authorities are willing to engage with IMI projects via a variety of means (10). In some cases, regulatory authorities are members of a consortium, in others they sit on advisory boards and in some cases they even suggest ideas to be considered for launch as a call topic in the IMI programme. The involvement of regulatory authorities covers a range of areas. IMI supports projects addressing challenges in the area of safety sciences in the hope of advancing more reliable tools for the accurate prediction of the safety of medicines. The SAFE-T^7^ project addressed the lack of biomarkers for the early detection of different forms of drug-induced toxicity (11). The eTOX^8^ project built a unique toxicology information database using legacy report from multiple sources including all pharmaceutical companies involved with the aim of developing better *in silico* tools that can better predict the toxicology of new compounds (12). Another area of interest to regulatory bodies has been the development of new tools and methods for benefit-risk assessment of medicines. PROACTIVE^9^ developed Patient Reported Outcome (PRO) tools that improve the capture of physical activity in relation to chronic obstructive pulmonary disease [COPD; (13)]. The PROTECT^10^ consortium has produced a set of recommendations for benefit—risk assessment processes and supporting tools (14, 15). Finally, clinical trial design and how to innovate in this area is a key challenge in attempting to speed up the drug development process. In IMI projects the regulatory authorities have been engaging and exploring what is possible in this domain. A good example is the EPAD^11^ project, where 10 pharmaceutical companies along with their public partners and other international bodies are collaborating to address the challenges involved in the selection of patient sub-groups, drug candidates, optimal end points, and statistical methodology (16, 17). The consortium members have been engaging and exploring with the regulatory authorities what is acceptable to them in this challenging endeavor.

To date IMI projects have built good interactions with regulatory authorities, however experience has shown that sometimes the projects leave this interaction until too late in the project to experience the full benefits of the interaction. IMI consortia working in the area of Alzheimer's such as EPAD or autism such as EU-AIMS^12^ (18, 19) have engaged with regulators at an early stage of the projects and this has resulted in very beneficial interactions in terms of ensuring the projects are on the right track and the buy-in of the regulators for their chosen approaches. In addition to interactions at the project level, the IMI programme office also organizes regular meetings with the EMA and FDA to hold strategic discussions on topics of common interest, underlining the importance of the regulatory environment for the work undertaken and the challenges been addressed by IMI-funded projects.

### How IMI Manages Call Evaluations and the Resulting Projects

The features of how the IMI programme works at the evaluation and project monitoring level is available on the IMI website. However, there are several key features that are worth consideration in order to understand how IMI differs from other funding programmes.

At the heart of how IMI works is the topic development process. While IMI is an equal partnership between the EC and EFPIA, with both founding partners contributing equal funding, it is the industry partners who determine the topics that IMI launches in its calls for proposals. Using the IMI2 Strategic Research Agenda (which provides a public health focused framework given it is based upon the WHO Health Priorities Report of 2013^13^), the industry partners come together and agree on where there they have a shared challenge and where working together will overcome the challenge more quickly than individual companies working alone. In addition, while agreeing the scientific focus of the topic, the companies also determine what resources they will commit to the eventual project. It is important to remember that the public funding provided to IMI goes to public beneficiaries identified through a competitive call process and that no public funding goes to industry partners. Based upon the industry resources identified, IMI then matches this with public funding and these two figures determine the overall budget included in the final call topic text. Call topic texts are consulted upon with the EC, SC and States Representative Group (an advisory body to the IMI GB) prior to approval by both founding partners via the GB.

Once a topic has been launched IMI invites applicant consortia composed of public entities to work together and submit a short proposal in response to a given topic text. Any entity may be part of an applicant consortium as long as they have a well-defined and non-redundant role within the consortium. These short proposals are then subject to a review by an independent panel of experts selected by IMI. This review is based on clearly defined, publically available criteria and the original topic text. The panel scores and ranks the proposals and only the top-ranked proposal is invited to the second stage. Only the top-ranked proposal is invited to the second stage, as at the second stage the successful applicant consortium is merged with the original industry consortium that prepared the topic text to form a completely merged full consortium. This full consortium then prepares a full proposal with detailed work plan, milestones and deliverables etc. Once again the full proposal is subjected to independent review by a panel of experts and this panel makes a go/no-go recommendation to the IMI GB. When the GB approves the recommendations the full consortium is invited to the granting stage of the process. During this phase the consortium agree a Consortium Agreement (CA) covering all aspects of project operations, access to data generated, IPR etc. The CA is the sole responsibility of the consortium partners; IMI does not participate in its negotiation, rather, once the CA is agreed IMI will then sign the Grant Agreement (GA) with the consortium.

Once the GA is signed then the project can start. During the lifetime of the project the IMI office staff monitor the projects very closely to ensure that the project is on track scientifically and that the project is being executed according to IMI's rules. Each project must submit a periodic report in which progress against the original work plan is checked and whether the public funding and industry contributions are being used in line with IMI rules. Although not obligatory under the H2020 rules, all IMI projects are subject to an interim review in which independent experts, usually headed by a member of the IMI SC, review the progress of the project and can make recommendations in case they identify issues. This is complemented at the end of the project with a close out meeting where project representatives come to the IMI office and explain what the project has achieved and what has been learnt.

### The Challenge of Forming an Applicant Consortium

Since the launch of IMI1's first call for proposals it has been recognized that the formation of applicant consortia presents a unique challenge for many researchers. Given the scope of many IMI topics applicant consortia need to be composed of multi-stakeholder, multi-disciplinary teams and the identification of these partners in different fields is not always straight forward. Many leading researchers already have established networks of peers in different countries working in different areas of research and these researchers have the advantage of having a pool of talent to draw on when it comes to consortium formation. More junior researchers or organizations, such as patient groups or SMEs, may not have well-established networks outside of their field of interest and therefore struggle to identify all the expertise that may be required to respond to an IMI call. In the interest of transparency and fair treatment, the IMI programme office is unable to assist potential partners to form consortia, adding to the challenge for some of those interested in applying in forming a suitable consortium. While there are different partnering search tools and different fora for researchers to network these are not always effective when trying to form a large consortium of diverse expertise at short notice. Therefore, it is important for anyone interested in collaborative programmes such as IMI that they establish their networks in advance of applying in the future. This challenge will persist in future programmes and may be exacerbated in programmes where the scope is envisaged to be broader than the current programme.

In order to help the formation of the consortia, IMI publishes its scientific priorities and draft topic texts as early as possible, sometimes several months in advance of official publication. However, this cannot fully compensate for having a well-established network of international collaborators.

The fact that industry plays a key role in determining the research priorities of the IMI programme is sometimes criticized as it is seen as giving too much control of the programme to industry partners. However, industry experiences the real challenges of drug development and the regulatory environment first hand, knows where they have failed and understands where the individual companies can collaborate. If we are to make real impacts upon the challenges within drug development processes, then we need to ensure that the challenges being addressed are relevant and will generate the desired impact. It is also important to remember that unlike other governmental or public led collaborations, IMI is not a co-funding model; rather it is a true collaboration. Unlike some other national PPPs with government agencies involved the industry partners are not seeking to buy a service or provide money for the execution of tasks. Within the IMI model, industry partners are fully engaged in the final project as they share the same responsibilities and obligations as the public partners. All industry partners sign the consortium agreement and also the grant agreement. During the project the industry partners have well-defined tasks and it is usual for work packages to have joint leadership with both public and private partners contributing work package leaders.

### IMI Progress in Numbers

By the end of 2018, under the two programmes 119 projects had been launched involving over 2,000 participations drawn from a wide range of stakeholders, and the portfolio is constantly growing^14^. The analysis of the data collected up to 31 December 2018^15^ shows that almost all the relevant priority areas in the IMI2 Strategic Research Agenda (SRA) have been addressed. For IMI1, as of the end of 2018 EUR 965.7 m of EU funding had been committed matched by EUR 965.1 m of in kind contributions committed by industry partners. For IMI2, EUR 655.6 m of EU funding had been committed matched by EUR 664.9 m of in kind contributions committed by industry partners.

The types of organizations involved in IMI at the end of 2018 include; 597 universities and academic institutions, 61 EFPIA members, 229 SMEs, 33 patient organizations, 29 regulatory authorities and 15 associated partners. The areas that have received the most funding to date include EUR 1.1 billion in the area of infectious disease (includes) AMR and vaccines, over EUR 300 m in the area of brain disorders and neurodegeneration, nearly EUR 250 m in the area of diabetes and metabolic disorders, EUR 214 m in drug discovery, EUR 142 m in cancer and EUR 126 m in the area of data knowledge and management.

IMI has launched projects covering a wide array of disease areas and challenges in the discovery and development of new medicines including infectious control (20), neurodegeneration (21, 22), cancer (23, 24), diabetes (25, 26), immunological disorders (27, 28), drug safety testing (11, 12), clinical trial design (29), and the use of real world evidence in drug development (30) to mention but a few. The outputs from the projects are many and varied and to date, the partners involved in IMI projects have generated 4,983 publications (with a normalized impact factor of 1.84, nearly double the EU average). The examination of the results shows that IMI2 projects have generated 16 assets that completed a significant milestone during the project lifecycle (vs. an overall target of 50 for the IMI2 programme), and if we look at both IMI1 and IMI2 programmes together, the analysis shows that 57 assets have completed a significant milestone so far. The definitions of “projects' asset” and “significant milestone” were meticulously defined. Examples of assets are tools, methodologies, processes, services, training materials, etc.; examples of significant milestones are key clinical trial phases, animal models, prototypes, commercialization, patents, publications, etc. A subset of IMI projects has managed to impact the regulatory framework and received acceptance by regulatory authorities: for IMI2 there are 7 completed procedures with 4 regulatory qualified opinions and 3 CE marks granted (vs. an overall target of 15 for the IMI2 programme). If we look at both IMI1 and IMI2 programmes together there are 15 complete procedures. Several new tools and processes generated by IMI2 projects have been implemented by the industry participants (examples of implementations are animal models, standards, biomarkers, SOPs, use of screening platforms, clinical trial networks, etc.). The data shows the take-up and utilization of 19 IMI2 project results (vs. an overall target of 50 for the IMI2 programme) and 122 results taken up by industry partners if both IMI1 and IMI2 programmes are considered together. Many tools and new methodologies have been published and information on tools available to researchers are available on the IMI website^16^. It is worth considering that many IMI2 projects have not yet reached their midpoint and there are many more projects to be launched. The data so far suggests that the programme is on track to meet its objectives and the projects are well on track to meet the expected targets for the key performance measurements of the initiative.

In such a short article it is impossible to do justice to all of the projects that have been launched under IMI2. This article will therefore focus on three areas: drug discovery, infectious diseases, and addressing unmet societal needs. It describes a couple of projects from each, demonstrating what can be achieved when different stakeholders collaborate at a scale which is in proportion to the challenge.

## Collaborating to Speed up the Discovery of New Medicines

Antimicrobial resistance (AMR) is a major global public health threat with bacteria becoming increasingly resistant to existing antibiotics. The rising mortality rates and extended hospitalisations for patients associated to this resistance is also translating into increasing treatment costs for health services. In 2018, the European Centre for Disease Prevention and Control (ECDC) released figures showing that 33,000 people die every year in Europe from infections that prove resistant to treatment, a number that is rapidly increasing. So there is an urgent need to discover and develop new anti-infectives, especially new antibiotics. However, not only is this scientifically challenging, but antibiotics have a low return on investment compared to other medicines, making them an unattractive area for drug developers. Indeed, as the use of new mechanism of action antibiotics will be limited by governments and health authorities in order to slow resistance acquisition, this area represents a true market failure and incentives for industry participation are thus warranted.

Through its New Drugs for Bad Bugs (ND4BB)^17^ programme, IMI has invested heavily in a portfolio of projects that address most of the challenges along the entire value chain of AMR R&D, facilitating collaboration and de-risking novel approaches (31). The first projects were launched in 2013 in response to the EU's action plan on AMR. The COMBACTE^4^ projects have now set up a network of hundreds of hospitals and laboratories to facilitate the conduct of pan-European clinical trials and studies (32, 33). The network is already being used extensively for a broad range of studies, including trials of potential new antimicrobials.

A very important element of the ND4BB programme is the discovery of new candidate antibiotics through the ENABLE project. ENABLE^18^ focuses on the discovery and pre-clinical stages of drug development, attempting to identify and accelerate the development of new compounds coming from both the public and private sectors. A key objective of ENABLE is to share the risk of developing new antibiotics between different partners, encouraging researchers to progress more compounds in this area (see ENABLE Call for Action—European Gram-negative Antibacterial Engine^19^). Compounds are sought from all researchers (from academia, SMEs, research organizations etc…) through an open call meaning anyone with an interesting molecule can apply. Spanning 13 countries, ENABLE has brought together 32 partners including 11 SMEs to help researchers overcome these thresholds. To date 70 programmes from SMEs and research organizations have been received and over 15 programmes have been integrated within its portfolio, of which 5 are currently running.

Recently, it reached the important of milestone of selecting as a potential antibiotic, apramycin, as a clinical candidate. Identified by researchers from the University of Zurich and further progressed in a university spin-out company Juvabis, the data package supporting apramycin's development was submitted to ENABLE and it was selected as a clinical candidate^20^.

In another example of how ENABLE is enhancing and speeding up drug discovery, compounds targeting Gram-negative infections were identified by Chris Schofield at the University of Oxford via the novel drug screening programme of another IMI project, the European Lead Factory's (ELF)^21^. After further refinement within the ELF, the compounds were submitted to ENABLE. When the compounds were reviewed by the ENABLE partners, they were found to have exciting potential and were included in the ENABLE programme with the hope of further development toward the clinic^22^. This access to drug discovery platforms and the rate of development is unprecedented and reveals the potential of IMI projects to help researchers rapidly go from a novel idea to potentially taking compounds into the clinic in the matter of a few years while addressing important areas of unmet need.

The European Lead Factory is not only limited to identifying new antimicrobials molecules, but is open to drug target programmes related to all human disease and all types of small molecules. It provides researchers unprecedented access to pharmaceutical company compound collections and high throughput screening (HTS) technology, allowing researchers to test their drug target ideas. The output from the project is a list of identified compounds for each target screened that can then be developed further by the researchers.

The project is composed of two arms. The first is the European Screening Centre housing the equipment and expertise to run the HTS services for the selected public projects. The second is a unique compound collection, the Joint European Compound Collection, coming from seven industry partners and complemented by compounds that have been sourced from European researchers. This compound collection is unique, being composed of compounds coming from the libraries of seven industrial partners and complemented by compounds coming from public partners. The over 500,000 compounds in the collection are not commercially available and cannot be found anywhere else in the world.

Through this unique platform, 88 new targets public programmes have been validated and screened, while nearly 6,000 qualified hits have been granted to public and private target owners, meaning that many researchers now have a valuable first step toward setting up their own new drug discovery programmes (34). Some of the results are already well-advanced. Dr Margit Mahlapuu from the University of Gothenburg had identified a target which could be used to reverse metabolic complications in type two diabetes (35). She submitted this target to ELF and the resulting screen identified a set of selective and potent small molecules which interfere with the target. Based on her research and armed with these new compounds, she created a spin-out company, ScandiCure, with the aim to develop the compounds further so that they could become a first in class anti-diabetic drug. The compounds have such promising potential that ScandiCure has now entered into an agreement with Servier for the further development of the compounds for the treatments for type 2 diabetes and the liver disease non-alcoholic steatohepatitis^23^.

In another example, Richard Mead of the University of Sheffield had grown frustrated with a lack of results after many attempts screening publicly available libraries and commercially sourced compound collections. He approached ELF with his target, a protein involved in oxidative stress that had been found to play an important role in motor neuron disease, Parkinson's disease and other neurodegenerative disorders. The results of the screens proved so interesting that Parkinson's UK decided to set up a “virtual biotech” company, Keapstone Therapeutics, based upon further developing the identified compounds^24^. Although there is still a long way to go, the compounds identified are very good starting points for developing potential Parkinson's treatments^25^.

Taken together it is clear that in the area of early drug discovery, there is much to be gained by collaborating, pooling resources, and expertise. Through the ELF and ENABLE projects, IMI is making resources available to the research community that are not available elsewhere, the results of which are kick starting new drug development programmes. These programmes offer patients and society the hope that new treatments may one day be found for currently difficult to treat conditions, or diseases where no treatment is available.

## Collaborating to Tackle Ebola

IMI launched two calls for proposals focused on Ebola in response to the outbreak that occurred in western Africa in 2014–2016. This outbreak was unprecedented in scale, with around 29,000 people infected and over 11,000 of them dying in the west African nations of Guinea, Liberia and Sierra Leone. As a result of these two calls, IMI now supports 12 projects addressing various aspects of Ebola. These include testing new vaccines, the implementation of clinical testing in outbreak areas, speeding up manufacturing routes, speeding up deployment of vaccines, as well as community engagement to educate and help with the uptake of the vaccines in the affected communities.

With no licensed vaccines available in 2014 there was an immediate need to bring forward safe and effective vaccines. IMI projects supporting vaccine development include EBOVAC1^26^, EBOVAC2^13^ and EBOVAC3^13^ as well as VSV-EBOVAC^27^, VSV-EBOPLUS^28^ and PEVIA^29^ among others. EBOVAC1, 2 and 3 focus on assessing the safety and tolerance of the prime boost Ad26.ZEBOV and MVA-BN-Filo vaccines. EBOVAC 1 supports Phase I trials testing the safety and tolerability of the vaccines in healthy volunteers in both Europe and west Africa. Further phase II and phase III trials aimed at speeding up the clinical development are being supported by both EBOVAC 1 and 2 projects, again, focused on west African communities. EBOVAC3 builds on this work and aims to run clinical trials in the very vulnerable children populations of Sierra Leone, Guinea and the Democratic Republic of Congo.

The data so far from the EBOVAC1 clinical trials demonstrate that the vaccines are safe and well-tolerated. Phase 1 findings so far reported indicate that Ad26.ZEBOV prime immunization readily induces an immune response which is enhanced further by MVA-BN-Filo, and that the Ad26.ZEBOV/MVA-BN-Filo heterologous prime-boost regimen induces durable immunity to the Zaire strain of Ebola, and that both the prime and boost are well-tolerated with a good safety profile (36).

The projects VSV-EBOVAC and VSV-EBOPLUS attempt to advance the development of the VSV-ZEBOV vaccine candidate. The recombinant vesicular stomatitis virus (rVSV) vaccine expressing the Zaire Ebola virus (ZEBOV) glycoprotein has been found to be efficacious following single-dose injection, with antibody responses sustained across dose ranges and settings (37). Finally, PEVIA aims to develop second generation vaccines that will be better suited to large scale vaccination programmes in sub-saharan Africa, specifically vaccines that will not require storage at low temperatures.

The challenges faced in trying to vaccinate populations in affected areas and ensure compliance with vaccination regimens are being addressed by the EBODAC^13^ project (38). The EBODAC project has developed communication strategies and tools to promote the acceptance and uptake of new Ebola vaccines. These include the development of many creative strategies including radio shows, drama performances and community meetings. Thanks to the efforts of EBODAC's team over 450 adults and children received both doses of the vaccine regimen in the Sierra Leone study and what has been learnt can be applied to current and future outbreaks (39).

To complement the projects focusing on vaccine development and running clinical trials in the field, IMI supports projects designing new or improving existing rapid diagnostic tests. A key feature of these tests is their ability to be used in the field where laboratory facilities may be minimal or non-existent (40). Rapid diagnostic test projects include Mofina^30^, FILODIAG^31^, EbolaMoDRAD^32^ and VHFMoDRAD^33^. The MOFINA project team built on the existing automated device “Alere q” to develop a portable assay system that can give an accurate diagnosis within 75 min. EbolaMoDRAD has developed technologies that allow Ebola samples to be handled safely outside of high containment laboratories. Finally, Filodiag has delivered a highly sensitive system that can deliver results in just 30 min. All these systems have been tested in the field and found to deliver reliable results much more quickly than previously used systems.

With new infectious diseases emerging all the time, these challenges can only be addressed through collaboration of all stakeholders, whether on the development of new treatments and vaccinations, their deployment in the field, or new diagnostic methods. In order to be prepared and able to cope with future outbreaks, all of society's stakeholders need to engage and work together.

## Collaborating to Address Unmet Societal Needs

While the IMI programme supports many projects directly addressing specific challenges in pharmaceutical R&D, it is also an appropriate vehicle to foster the necessary collaboration to overcome some wider societal challenges in areas such as pediatric medicines or the use of medicines in pregnancy. Based on previous assessments, only 30% of marketed drugs in Europe and worldwide include a pediatric authorization, and <50% of authorized medicines commonly used in children have been properly tested in this population (41, 42). This rate drops to 10% in the vulnerable patient population in neonatal intensive care units (43). In order to address this deficit, the “Pediatric Regulation” came into force in the EU on 26 January 2007 with the objective of improving the health of children in Europe by facilitating the development and availability of medicines for children aged 0 to 17 years^34^. A review of the landscape in 2017^35^ found that there had been an increase in medicines for children in many therapeutic areas in the last 10 years, most notably in rheumatology and infectious diseases. However, it also found little progress had been made in diseases that only affect children, or where the disease shows biological differences between adults and children, particularly rare diseases.

One of the reasons for the slow progress is that running clinical trials in children is very difficult and this has inspired IMI to launch the conect4children^36^ (c4c) project to create a sustainable, integrated, pan-European collaborative pediatric network. The c4c consortium aims to create a network that will deliver high quality “regulatory grade” clinical trials (phase I to IV) from different sponsors, in different therapeutic areas, and across all age groups. The viability of the network will be tested in pilot studies from industry and non-industry partners. The first four pan-European pediatric studies will be conducted by academic institutions and will generate data on high priority medicines commonly used in babies, children and young people in Europe^37^. In addition to facilitating the testing of new medicines, the new network will also provide expert advice and ensure that the voices of young patients and their families are heard to guarantee the conduct of feasible, innovative and scientifically sound pediatric clinical trials. It should be noted that c4c is already attracting attention internationally and plans are already in the making for creating a productive interface with a similar initiative in the US called the Institute for Advanced Clinical Trials for Children (I-ACT)^38^.

Another area of current unmet need is in the information available to guide decision making for the safe and effective use of medications during pregnancy and breastfeeding (44, 45). Pregnant and breastfeeding women with chronic diseases may need to continue their medicines to treat their conditions in order to prevent irreversible damage to their health and the health of their unborn child that may be caused in some situations by the disease itself when the treatment is stopped. However, prescribing information leaflets generally lack clear information to inform decision-making, meaning that practitioners and patients alike are unable to make informed decision on treatment approaches.

Pregnant and breastfeeding women have been purposively excluded from clinical trials and currently a common source of post-authorization safety data on medicines in pregnancy comes from product-specific pregnancy registries. According to a Food and Drug Administration (FDA) review based on 59 pregnancy registries, only a minority (12%) generated data that was used to inform the label to adequately advise patients and healthcare providers (HCPs). Where data was not used to inform the label, this was usually due to the inadequate recruitment of subjects and a lack of internal comparator groups (46). Hence, many compound-specific pregnancy registries close several years after initiation. In addition, there is no consistent standard of data quality (data collection and analytical methods) recognized as warranting inclusion in product labels. The situation is even worse concerning breastfeeding.

IMI launched the ConcePTION^39^ project to address the major public health concern for a robust, evidence-driven approach to define the standards for generating data on medicines used during pregnancy and breastfeeding. This is a unique endeavor gathering lead experts in the field from both public and private sectors that will build an ecosystem for better monitoring and communicating of medication safety in pregnancy and breastfeeding. To change current practices, the project sets out to define more timely and efficient data collection and analytical approaches compared to current pregnancy registries. It is hoped that this improved information will enable HCPs and pregnant patients to make informed decisions regarding medication use, and enhance their care. The project also aims to support more basic research approaches such as the development of better animal lactation models that more closely reflect human lactation physiology. The intention is also to build a Europe-wide breast milk biobank and support an analytical centre for the analysis of drug concentration in milk so that samples are more readily available and analytical methods can be improved.

In the two examples described there is a key healthcare challenge for society and the scientific questions underpinning these can only be addressed by different actors analyzing the problem, sharing their results, and agreeing the way forward. IMI plays an important role by bringing these different stakeholders together to collaborate with the aim that the results will benefit children and their mothers. The ability to mobilize many different stakeholders and bring together data at a scale not achieved before should ensure that the results of the collaborations are transformative for the respective fields. The fact that these collaborations occur as part of a public-private partnership ensures that there is a fast feedback mechanism for results to flow back to the pharmaceutical companies. It also provides a neutral interface with which the regulators can interact so that the results can help inform regulatory science.

## Mobilization of Other Stakeholders

Over the past 10 years we have witnessed an evolution in the challenges been addressed by the IMI programme. It has also become apparent that other stakeholders beyond the pharmaceutical industries are needed if these challenges are to be effectively addressed. During the IMI1 programme several non-EFPIA industries participated however the rules of IMI1 did not allow IMI to match the in-kind contributions provided. Under IMI2, the in-kind contributions of non-EFPIA organizations can be matched by public funding as long as the entities agree to become Associated Partners of the IMI2 programme and be bound by the same rules and obligations of all other partners participating in projects. In this way additional non-EFPIA contributions can be brought onboard and be leveraged by public funding. This change makes the programme much more attractive for other funders and companies to participate. Indeed, IMI has become a magnet, attracting new partners who understand the value of becoming involved in these collaborative projects if advances in many challenging areas of public health are to be achieved. As of June 2019, 31 entities had become Associated Partners to IMI2, participating in 44 projects (launched or in preparation) and are contributing nearly EUR 180 million to the programme. IMI2 Associated Partners are listed in Table 1. On top of this, many more organizations, primarily from other industries, are aligning with EFPIA so that they can contribute to specific IMI projects as EFPIA “Partners in Research”^40^. While the participation of APs may help boost the funding available for the programme it is important that APs are fully aligned with the objectives of the IMI2 programme and share the collaborative vision of combining knowledge and expertise so that challenges can be overcome much more effectively.

**Table 1:** IMI2 JU Associated Partners as of June 2019.

**Associated partner**	**Project/topic**
Accelerate Diagnostics	VALUE-Dx
Autism Speaks	AIMS-2-TRIALS
Autistica	AIMS-2-TRIALS
BD Switzerland Sarl	VALUE-Dx
Bill and Melinda Gates Foundation (BMGF)	PERISCOPE Call 15, Topic 08—AMR (Pillar B)
Bio-rad Laboratories	VALUE-Dx
Cepheid Europe	VHFMoDRAD
CHDI Foundation	Call 15, Topic 06—Digital Endpoints
Children's Tumor Foundation (CTF)	Call 15, Topic 01—Integrated Research Platform
Datapharm	Call 18, Topic 03—Improving Patient Access: Integrated digital health
Diamond Light Source	Call 17, Topic 02—Open Access Chemogenomics Library
European Hematology Association	Call 18, Topic 06—Supporting the development of engineered T-Cells
International Diabetes Foundation	HYPO-RESOLVE
Invicro	Call 15, Topic 05—Platforms supporting Synaptopathy Drug Discovery
JDRF	INNODIA BEAT-DKD HYPO-RESOLVE Call 15, Topic 04—Emerging Translational Safety Technologies Call 17, Topic 01—Optimizing Future Obesity Treatment Call 18, Topic 02—Health Outcomes Observatories
KTH Royal Institute of Technology	Call 17, Topic 02—Open Access Chemogenomics Library
Leona M. And Harry B. Helmsley Charitable Trust	INNODIA HYPO-RESOLVE
McGill University	Call 17, Topic 02—Open Access Chemogenomics Library
Medicines for Europe	Call 18, Topic 03—Improving Patient Access: Integrated Digital Health
Medicines for Malaria Venture (MMV)	ESCulab
Obesity Action Coalition	Call 17, Topic 01—Optimizing Future Obesity Treatment
Ontario Institute of Cancer Research	Call 17, Topic 02—Optimizing Future Obesity Treatment
Parkinson's UK	NEURONET PD-MitoQUANT PD-Mind Call 15, Topic 06—Digital Endpoints
Simon Foundation Autism Research Initiative (SFARI)	AIMS-2-TRIALS
Software AG	RADAR-AD
Springworks Therapeutics	Call 15, Topic 01—Integrated Research Platform
T1D Exchange (formerly Unitio)	HYPO-RESOLVE Call 17, Topic 01—Optimizing Future Obesity Treatment
TB Alliance	Call 15, Topic 01—Integrated Research Platform Call 15, Topic 08—AMR (Pillar B)
Trial Nation	Topic 02—Health Outcomes Observatories
University of Dundee	Call 15, Topic 08—AMR (Pillar B)
Wellcome Trust	VALUE-Dx

## Some Lessons Learned

From experience to date, it is clear that IMI works as a collaborative platform allowing different stakeholders to work together to resolve problems and address issues that single entities are unable to address alone no matter their size or wealth. However, it is also clear that when mobilizing many different actors to tackle a diverse range of different problems, whether in scientific research or healthcare, this raises its own challenges. The first obvious challenge is how to get different organizations to work together. When IMI was set up many doubted whether pharmaceutical companies would engage with such a platform and collaborate with each other. Experience has shown that not only have pharmaceutical companies engaged with the programme, but they recognize that for certain challenges, this is the only way to advance scientific knowledge. When the programme began there was a reluctance from some parties to engage as it was not clear what would be the benefit for public entities collaborating so closely with industry consortia. Experience has shown that the collaborations are very fruitful and that public partners can generate the good quality publications they need as well as accessing data and resources simply not available elsewhere in the public domain. In fact many of the networks established in IMI projects are sustained beyond the IMI funding period leading to long lasting collaborations between different sectors.

Today many other stakeholders, whether they be patients, researchers or regulatory agencies, are eager to engage with IMI and participate in its projects. They recognize the value of the platform for the sharing of different experiences, knowledge and approaches. It is the role of IMI as a neutral platform to encourage this engagement and to allow stakeholders to build the trust in each other that will then facilitate collaboration. The neutrality of the IMI Programme Office is a key foundation upon which successful projects are built.

The monitoring of these projects both from a scientific and an administrative point of view can also be very challenging. Therefore, it is important that projects are well-managed and in particular have leadership that enables the project partners to stay on track. Large projects can also be scientifically unwieldy resulting in difficulties for the consortia to change the course of the research once the objectives have been set. Although there is flexibility in the IMI programme to allow changes when scientifically justified, it is sometimes difficult for IMI projects to respond to rapid advances in knowledge as quickly as they would perhaps like.

The projects cited in this review operate under the same sets of legal rules as other FP7 or Horizon 2020 projects. Whether it is researchers clinically testing an Ebola vaccine in the field, or a HTS project identifying new compounds as the starting points for medicines, or researchers building biobanks to help understand the exposure to medicines in breast milk, the same legal framework applies to all. This ensures consistency across all projects and in the majority of cases the frameworks are well-constructed and appropriate so the projects operate very well. However, sometimes the IMI platform could achieve even more if it were able to mobilize additional resources not in the public domain or apply the rules in a more flexible way than is currently allowed. Hence this lack of flexibility, where “one size fits all” can sometimes hinder the adoption of radically innovative approaches.

One of the questions often asked is what is the impact of the IMI platform on the health of European citizens; after all, a large amount of public funding is being invested through the programme. The IMI Programme Office invests a lot of energy in defining appropriate measures of impact and progress. Some measures are relatively straight forward to collect, such as bibliometrics, patents, tools developed etc. However, tracking impacts outside the project becomes increasingly difficult, especially after a project has finished, and the further we move from the domain of pre-competitive research into the healthcare space. Many of IMI's projects tackle challenges in the early pre-competitive space. Given that the time to develop new medicines can be 10–15 years, this is a long time to wait for an impact to appear eventually in the healthcare system. Also, healthcare is a very complex system where medicine, society and politics come together, meaning that attribution of a given project result to a given impact is incredibly difficult.

A great example of this is the impact coming from IMI's PROactive project. This project had as its main objective to obtain a regulatory accepted patient reported outcome (based on physical activity) in the context of new treatments for COPD (chronic obstructive pulmonary disease). Although this goal was indeed achieved, the qualification opinion from the EMA was given 2 years after the official end of the project^41^. Many other projects will see the impact of their work materialize many years after the funding cycle.

## What Next for Collaboration in the Healthcare Space?

As the population ages, the burden of disease is increasing with the result that healthcare costs continue to rise with no sign of slowing. Beyond an aging population, infectious diseases are becoming increasingly resistant to treatment with new infectious diseases emerging. These are just two examples of the challenges we will continue to face. We are unable to avoid the conclusion that current and future challenges in healthcare can only be addressed by all stakeholders working together. Therefore, we need to find the mechanisms to harness the talents of the different stakeholders and bring them together to work toward achieving a single goal. Healthcare is an area where collaboration is inescapable, and platforms that allow the problems and issues to be addressed in a neutral space will be essential. An essential first step for these future collaborations will be the building of trust between industries and organizations that may not currently be used to working together. This is particularly relevant when industries with different cultures, different business models and different objectives are being asked to work together for the first time. This is equally true for the different governmental agencies and regulators that will be required to work together as healthcare challenges are tackled.

An ongoing debate is whether public investment in research and in PPPs, such as IMI, in particular demands a quid pro quo with regards to pricing and the affordability of new treatments derived from IMI-funded research. IMI was originally set up to address key challenges in the pre-competitive space of drug development. Recently several IMI projects have been launched supporting the late stage development of assets in areas of high societal need such as AMR and Ebola. In these cases, the approach of the IMI programme has been one of sharing the risk in these difficult areas to ensure that society benefits from having new anti-infective agents and vaccines that might otherwise not be developed. If the programme is successful in making R&D processes more efficient it follows that these efficiencies should be reflected somehow in the affordability of medicines. The questions related to public investment in research and the eventual pricing of medicines is a legitimate one, but one which IMI was not designed to address. This key question will need careful consideration in the planning and design of future collaborative approaches.

Ultimately, researchers and clinicians working in the drug discovery and medicines space are motivated by the desire to understand and tackle disease. We need patients as partners in this cause to ensure that the work that is undertaken is relevant to their needs and we can deliver the treatments that they are waiting for. Under IMI2, we have already taken steps to better involve patients in our programme through the launching of the ‘IMI pool of patients experts’ where patients can help us by providing input into our strategy, our documents, our reviews, etc.^42^. And we can help them understand better and become more involved in scientific research as equal partners. Patients are an essential partner in all future collaborations.

As mentioned above, solutions to healthcare challenges involve disciplines and industries beyond the pharmaceutical industry and in some cases traditional healthcare providers. New approaches are needed to engage with ICT providers, artificial intelligence industries and those platforms generating personal data so that a more holistic approach is adopted for treating individuals. The issue of ensuring data quality should not be underestimated in these new approaches. A large amount of resources will be needed to ensure the quality, interoperability and standardization of the different types of healthcare-related data that are available and will be generated in the future. In IMI2 we already have a programme called Big Data for Better Outcomes^43^ focused on bringing real world data into the programme and using it to help provide solutions. Future programmes will have to redouble efforts in these areas as we will soon be unable to afford the continuously growing burden of chronic diseases. It will be necessary to use new technologies and approaches to improve prevention, and to speed up the early detection of chronic diseases.

We also need to recognize and accept that to deliver change in the healthcare space is very challenging. Healthcare systems have evolved over a long time and the actors involved are under intense political and societal scrutiny, so delivering change is not an easy task and should not be underestimated. If it is expected that future collaborations will be judged on their ability to drive this change, then we need to invest in ways in which to measure the expected changes in what is a highly dynamic and complex system. Therefore, the measures by which future collaborations are judged will have to be carefully chosen otherwise it may not be possible to assess whether the collaboration had any impact at all, never mind the desired impact.

In addition, in complex systems such as healthcare the goals of future collaborations need to be focused. Sometimes in the desire to keep all stakeholders and sections of society engaged, there is a tendency to promise that a programme or a project will be able to do everything. If those behind a future collaborative platform follow this approach, then it is highly likely that the programme will fail simply because the impact it might have had will be so diluted to the point that it may be impossible to demonstrate any impact at all. Therefore, it is essential that future collaborations are focused with clear, achievable objectives.

Since its inception, IMI has been a highly successful experiment in collaboration across a range of different and challenging areas. Not everything has gone smoothly; some approaches have been very successful, while others are less well-adapted to the current framework. It is important that these learnings are incorporated into any future programmes. At the dawn of this digital age in which data and artificial intelligence are becoming ever more important, the boundaries between traditional disciplines are blurring and falling away. Future collaborations will involve entities and disciplines currently not involved in healthcare. Against this dynamic backdrop the challenge for us all is to ensure that future collaborations in healthcare are fit to address these new challenges and deliver the more effective, safer medicines that patients deserve.

## Author Contributions

All authors listed have made a substantial, direct and intellectual contribution to the work, and approved it for publication.

### Conflict of Interest

HL and PM are employees of IMI, the IMI programme office has no role in the day to day management of the projects or the scientific research undertaken by the projects. The authors declare that the research was conducted in the absence of any commercial or financial relationships that may be construed as a potential conflict of interest.

## Footnotes

^1^https://www.imi.europa.eu/about-imi/mission-objectives

^2^http://btcure.eu/

^3^https://www.imi.europa.eu/apply-funding/general-overview/intellectual-property

^4^https://www.eupati.eu/

^5^https://imi-paradigm.eu/

^6^https://www.imi-prefer.eu/

^7^http://www.imi-safe-t.eu/

^8^http://www.etoxproject.eu/

^9^https://www.imi.europa.eu/projects-results/project-factsheets/pro-active

^10^http://www.imi-protect.eu/

^11^http://ep-ad.org/

^12^https://www.eu-aims.eu/#

^13^https://www.who.int/medicines/areas/priority_medicines/MasterDocJune28_FINAL_Web.pdf?ua=1

^14^https://www.imi.europa.eu/projects-results

^15^IMI Annual Activity Report 2018. Available online at: https://www.imi.europa.eu/sites/default/files/uploads/documents/reference-documents/AAR2018_final.pdf

^16^https://www.imi.europa.eu/projects-results/catalogue-project-tools

^17^https://www.imi.europa.eu/projects-results/project-factsheets/nd4bb

^18^http://nd4bb-enable.eu/enable-portfolio

^19^ENABLE Call for Action - European Gram-negative Antibacterial Engine https://www.youtube.com/watch?v=yUS607nwgIQ&feature=youtu.be (accessed July 23, 2019)

^20^ENABLE Juvabis Press Release, 2018: Tackling Antimicrobial Resistance: ENABLE Selects First Clinical Candidate available from http://nd4bb-enable.eu/press-release (accessed July 23, 2019).

^21^http://www.europeanleadfactory.eu/

^22^ELF Press release, November 2016: Promising antibiotic programme gets European boost https://us12.campaign-archive.com/?u=d2300afdcb71d3d71dfe70fbd&id=57013abee0; http://www.europeanleadfactory.eu/node/52 (accessed July 23, 2019).

^23^Servier press release, April. 2018. “Servier and Scandicure Enter into Agreement to Conduct Research in the Field of Metabolic Diseases.” https://servier.com/wp-content/uploads/2018/04/PR-Servier-Scandicure_2018.04.03.pdf

^24^https://www.europeanleadfactory.eu/news-events/virtual-biotech-company-launched-battle-parkinson%E2%80%99s

^25^Parkinson's UK press release March 2017. Keapstone Therapeutics launched in world-first partnership to develop new drugs for Parkinson's. https://www.parkinsonsvirtualbiotech.co.uk/single-post/2017/03/08/Keapstone-Therapeutics-launched-in-world-first-partnership-to-develop-new-drugs-for-Parkinson%E2%80%99s (accessed August 08, 2019).

^26^https://www.ebovac.org/

^27^http://www.vsv-ebovac.eu/en/home.html

^28^https://vsv-eboplus.eu/

^29^http://www.pevia-ebola.eu/

^30^https://www.imi.europa.eu/projects-results/project-factsheets/mofina

^31^http://www.filodiag.eu/

^32^http://www.ebolamodrad.eu/

^33^https://vhfmodrad.eu/

^34^Regulation (EC) No 1901/2006 of the European Parliament and of the Council of 12 December 2006 on medicinal products for pediatric use.

^35^State of Pediatric Medicines in the EU 10 years of the EU Pediatric Regulation COM (2017) 626.

^36^https://conect4children.org/

^37^https://conect4children.org/news/press-release-launch-of-the-non-industries-study/

^38^https://www.iactc.org/

^39^https://www.imi.europa.eu/projects-results/project-factsheets/conception

^40^https://www.efpia.eu/about-us/membership/#tab3

^41^https://www.ema.europa.eu/en/documents/regulatory-procedural-guideline/qualification-opinion-proactive-chronic-obstructive-pulmonary-disease-copd_en.pdf

^42^https://www.imi.europa.eu/get-involved/patients/imi-pool-patient-experts

^43^https://www.imi.europa.eu/projects-results/project-factsheets/bd4bo
